# *Ex vivo* real-time monitoring of volatile metabolites resulting from nasal odorant metabolism

**DOI:** 10.1038/s41598-019-39404-x

**Published:** 2019-02-21

**Authors:** Aline Robert-Hazotte, Rachel Schoumacker, Etienne Semon, Loïc Briand, Elisabeth Guichard, Jean-Luc Le Quéré, Philippe Faure, Jean-Marie Heydel

**Affiliations:** Centre des Sciences du Goût et de l’Alimentation, UMR 6265 CNRS/1324 INRA/Université de Bourgogne Franche-Comté, 9 boulevard Jeanne d’Arc, F-21000 Dijon, France

**Keywords:** Olfactory system, Analytical chemistry

## Abstract

Odorant-metabolizing enzymes are critically involved in the clearance of odorant molecules from the environment of the nasal neuro-olfactory tissue to maintain the sensitivity of olfactory detection. Odorant metabolism may also generate metabolites *in situ*, the characterization and function of which in olfaction remain largely unknown. Here, we engineered and validated an *ex vivo* method to measure odorant metabolism in real-time. Glassware containing an explant of rat olfactory mucosa was continuously flushed with an odorant flow and was coupled to a proton transfer reaction-mass spectrometer for volatile compound analysis. Focusing on carboxylic esters and diketone odorants, we recorded the metabolic uptake of odorants by the mucosa, concomitantly with the release of volatile odorant metabolites in the headspace. These results significantly change the picture of real-time *in situ* odorant metabolism and represent a new step forward in the investigation of the function of odorant metabolites in the peripheral olfactory process. Our method allows the systematic identification of odorant metabolites using a validated animal model and permits the screening of olfactory endogenously produced chemosensory molecules.

## Introduction

Following inhalation orthonasally or exhalation retronasally, odorant molecules travel in the airflow of the nasal cavity to reach the sensory olfactory mucosa (OM)^1–3^. This allows the deposition of a significant amount of molecules in the mucosa that activate dedicated olfactory receptors, triggering the olfactory signal^4^. The OM is an epithelial structure that lies at the interface between airborne compounds and the whole organism, including the brain (*via* olfactory neurons). The residence time of odorants in the OM environment affects their bioavailability, which is critical regarding (i) activation *vs* the saturation of olfactory receptors, (ii) potential toxicity for the OM and (iii) distribution of odorants to the brain or rest of the body. Odorant bioavailability is under the control of perireceptor events, including the action of odorant-metabolizing enzymes (OMEs) involved in odorant biotransformation^5^. OMEs are xenobiotic-metabolizing enzymes involved in detoxification by the enzymatic deactivation of chemicals and conversion into easily eliminable hydrophilic metabolites^6^. Odorants are substrates of these enzymes, which are highly expressed in olfactory tissues (and in similar concentrations to those in the liver, if measured on a per-cm^2^ tissue basis)^7–10^. In addition to some studies conducted with insects^11–13^, recent studies have demonstrated the function of perireceptor OMEs in odorant biotransformation catalysis in vertebrates, as well as olfactory signal modulation and, consequently, olfactory perception itself^14–18^. We recently demonstrated that odorant-odorant competitive interactions exist at the enzyme level for the odorant 2-methylbut-2-enal (the mammary pheromone) in rabbits. Conceptually, if two odorants compete with the same enzyme in the OM, one odorant is metabolized at the expense of the second that accumulates and activates more receptors. Accordingly, in rabbit pups, such metabolic competition with a competitor odorant strikingly enhanced perception of the mammary pheromone^14^. Enhancement of the signal consecutively to odorant accumulation was also observed in rats using electrophysiology after exposure to OME chemical inhibitors^18^. However, the odorant signal rapidly decreases due to the saturation of the receptors and neuronal adaptation. Nagashima and Touhara (2010) showed that, after exposing mice to odorants, their metabolites were detected in the mucus washed out from the nasal cavity. Moreover, following *in vivo* treatment with the corresponding OME inhibitors, they observed significant changes in both the activated glomerular pattern in the olfactory bulb and olfactory perception in response to odorants. The authors proposed that metabolites, by potentially interacting with receptors, might be involved in the perception initiated by the parent odorant^16,17^. Additionally, in a single study in humans, the presence of odorant metabolites has been demonstrated by an atmospheric pressure chemical ionization (APCI) ion source in exhaled breath after odorant inhalation^17^. This direct-injection mass spectrometry technique is very suitable for real-time analysis of volatile molecules from biological environments^19^.

Despite these advances, the significance of OMEs in the process of olfaction remains debatable because few aspects are known about the enzymatic mechanism and its ability to generate odorant metabolites, especially under experimental conditions directly focusing on the tissue involved: the neuroepithelium. We previously set up and validated an automated *ex vivo* headspace gas chromatography (GC) method^20^. Odorants in the gas phase were injected into the headspace of a vial containing a fresh explant of OM, and then the headspace was sampled and injected into the GC for analysis. We measured a decrease in the odorant concentration, which accounts for its metabolism by the tissue explant under near-biological conditions^20^. Using the same *ex vivo* experimental conditions, after a single injection of the odorant in the headspace, we used direct-injection proton transfer reaction-mass spectrometry (PTR-MS) to monitor the metabolism of ethyl acetate and the corresponding ethanol metabolite synthesis in real-time^21^. However, this device only allowed discontinuous recording that started from 10 seconds and was affected by a slow headspace equilibrium due to the experimental conditions (odorant injection in a 20-mL vial).

Here, we developed and validated an innovative technical approach based on continuous direct-injection analysis mass spectrometry using PTR-MS. It was designed to continuously deliver odorants to the OM explants to allow real-time monitoring of the headspace for both odorant uptake and the release of volatile metabolites (resulting from odorant metabolism). The method was successfully applied mainly to two class of odorants (carboxylic ester and diketones) that are structurally different, not sensorially related to humans, and involve different metabolizing enzymes, carboxylesterases and ketone reductases, as confirmed using specific inhibitors. To better understand the role of perireceptor enzymatic mechanisms in olfaction, our work stimulates potential research about the identification of volatile odorant metabolites and study of their potential impact on olfactory perception. Additionally, our results provide new insights into the *in situ* olfactory metabolism of odorants that update our understanding of nasal volatile metabolism that has been conceptualized in the olfactory physiologically based pharmacokinetic models^22,23^ developed in nasal toxicology.

## Results

### Real-time *ex vivo* odorant metabolism analysis method

**Figure 1:**
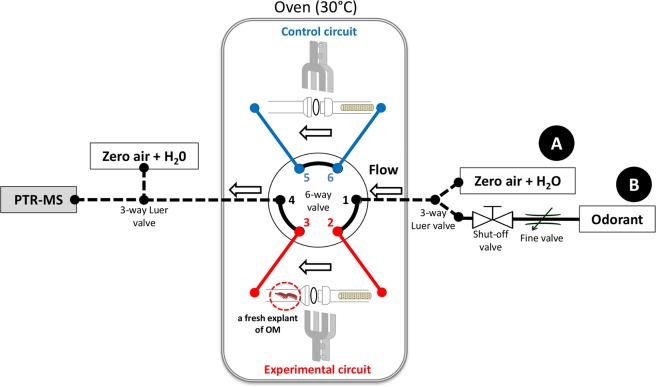
Experimental device for the real-time on-line measurements of the *ex vivo* odorant metabolism in OM. A 6-way valve connected to a PTR-MS instrument was implemented to allow odorants delivery in two independent circuits; the control and the experimental circuit containing a fresh explant of OM in glassware. The whole system is enclosed in a thermostated oven (30 °C). A known concentration of gaseous odorants can be continuously delivered by the fine valve from a gas bag B and the flow was monitored by the PTR-MS instrument allowing the real-time analysis of odorants and metabolites produced.

To investigate the metabolic capacity of *ex vivo* OM toward odorants in real-time, a continuous direct-injection mass spectrometry method was developed. It comprised the implementation of a 6-way valve creating a two-way circuit connected to a PTR-MS instrument that allowed direct odorant delivery independently above the biological material contained in a glassware in the first experimental branch, with the second branch serving as a control (Fig. 1). Equivalency of the experimental and control circuits was first checked for the flow rate and PTR-MS signal measurements.

### Real-time metabolism of ethyl acetate (EA)

**Figure 2:**
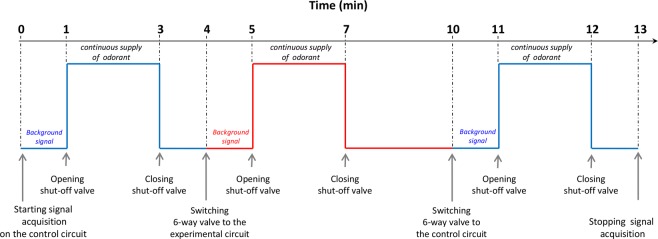
Schematic representation of the experimental protocol used for the real-time on-line measurements of the *ex vivo* odorant metabolism in OM by PTR-MS. Two independents circuits, the control (blue line) and the experimental circuit (red line) were used alternatively to measure the *ex vivo* odorant metabolism in OM. The 6-way-valve allowed to switch to one circuit to another without stopping the PTR-MS acquisition signal and the shut-off valve allowed to deliver or not the odorant in the active circuit.

**Figure 3:**
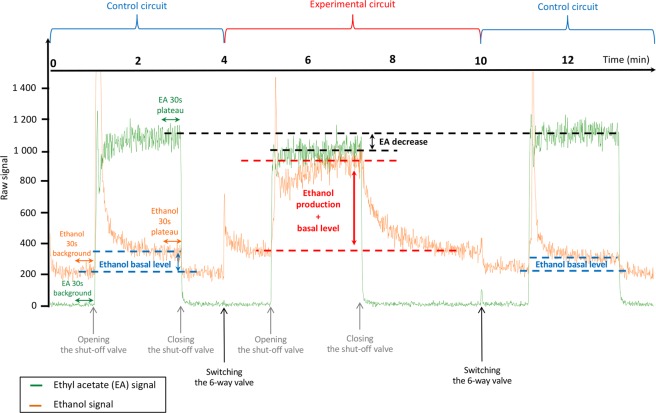
Example of raw data corresponding to real-time *ex vivo* ethyl acetate metabolism in OM by PTR-MS measurements and explanation of the main parameters. At the beginning of the acquisition, the PTR-MS recording focused on the EA and ethanol corresponding background signals. Throughout the experiment, only the odorant EA was supplied in the circuits. The opening of shut-off valve resulted in a characteristic increase of the signals before stabilization of the EA signal and the ethanol signal was slightly increased to reach its basal value in the whole device (blue double arrow). The closure of shut-off valve allowed to recover the background signals. The same signal modification was observed when the 6-way valve was switched. EA decrease (black double arrow) and ethanol production (red double arrow) in presence of OM were measured by comparison of the corresponding signals between the control and the experimental circuits.

As a feasibility test, the first assay was conducted using the carboxylic ester odorant EA because its metabolism that involves carboxylesterase enzymes and leads to its metabolite ethanol is well documented^21,24–26^. Moreover, monitoring of the reaction with PTR-MS had been demonstrated to be possible by a previous study using discrete sampling of the headspace above the OM contained in vials^21^. Blanks of the headspace above the OM in the glassware were recorded using humidified zero-air (gas bag A; Fig. 1) and were not found to be different from the control. According to the methodology presented in Fig. 2 and described in the Methods section (on-line measurements), a known concentration of gaseous EA (30 µg/L in the gas phase of gas bag B; Fig. 1) was continuously injected above a fresh explant of OM in the experimental circuit to compare with continuous injection in the control circuit without OM. Additionally, regarding the enzymatic mechanism responsible for this odorant metabolism, we performed metabolism analysis against two “enzymatic controls”: (1) inactivation of any enzymatic activity toward EA by heating the OM and (2) treatment of the OM with the bis (4-nitrophenyl) phosphoric acid inhibitor (BNPP) for specific inhibition of the carboxylesterase enzymes involved in EA metabolism. BNPP is an organophosphate that acts as an irreversible inhibitor of carboxylesterases, resulting in the generation of a stable phosphate ester covalently attached to the catalytic serine reside present within the enzyme active site^27,28^. Therefore, each experiment was independently performed against controls evaluated in the same spatio-temporal experimental conditions. Figure 3 presents an example of raw data corresponding to the real-time *ex vivo* OM metabolism of EA and ethanol production by PTR-MS measurements. The method starts by the acquisition of the EA and ethanol background signals. Throughout the experiment, only the odorant EA was supplied in the circuits. The opening of the shut-off valve resulted in a characteristic increase in the signals before stabilization of the EA signal and the ethanol signal was slightly increased to reach its basal value in the whole device (control circuit; blue double arrow). The closure of the shut-off valve allowed the recovery of the background signals. A burst of ethanol was observed when the 6-way valve was switched and when the opening of the shut-off valve allowed EA exposure to the experimental and control circuits. This burst is likely due to the flow variation at the valve opening together with unfortunate contamination of the shut-off valve with ethanol, precluding any conclusion to be made about the very first moment of the recording. Although zero-air was used as the carrier gas flow, the presence of a low background of ethanol in control assays may have come from the environment^29^, as well as the bags or parts of the system. EA decrease (black double arrow) and ethanol production (red double arrow) in the presence of OM were measured by comparing the corresponding signals between the control and experimental circuits. Each experiment was analyzed against controls performed under the same experimental conditions.

**Figure 4:**
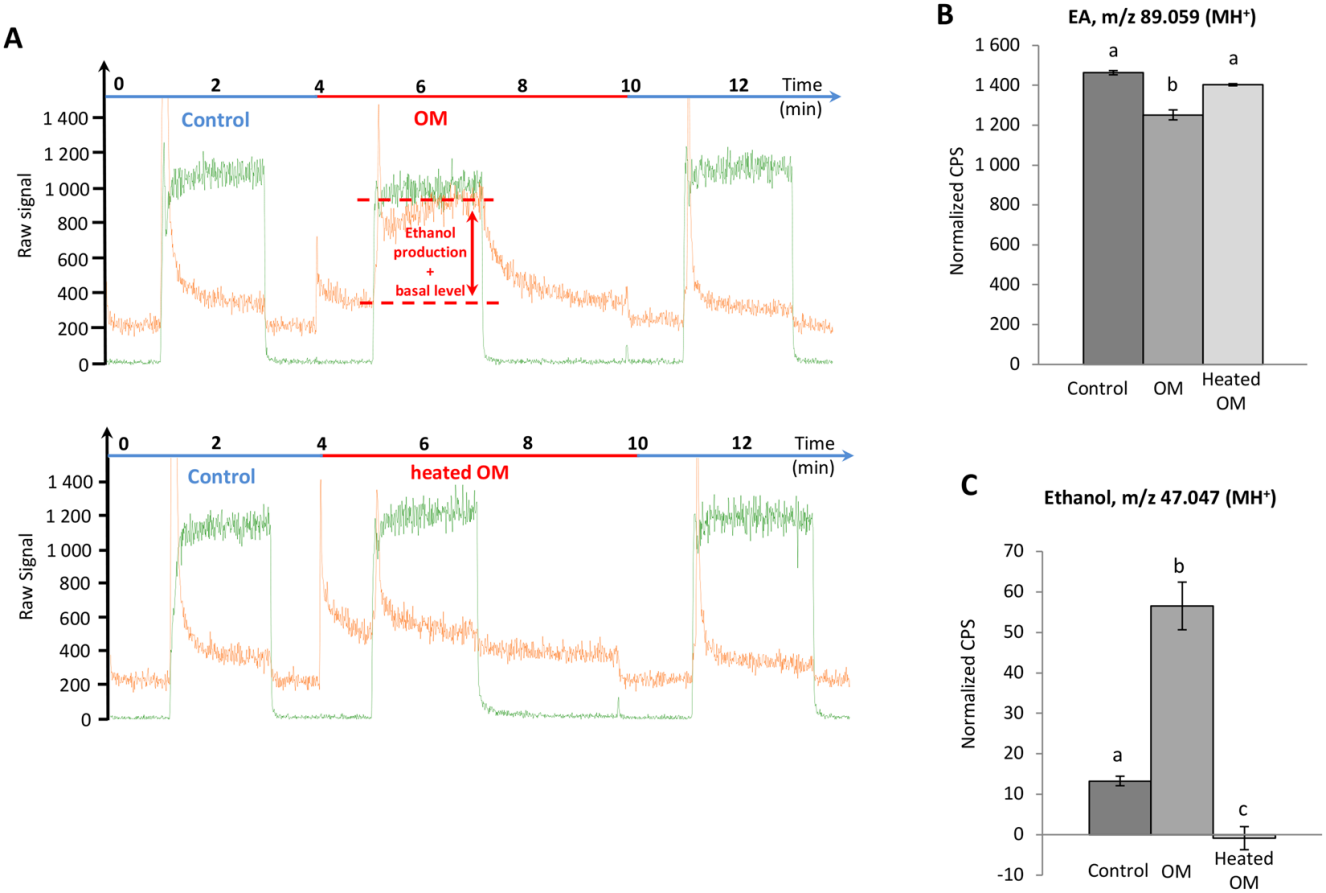
Real-time *ex vivo* OM metabolism of ethyl acetate and ethanol production by PTR-MS measurements: effects of enzyme denaturation by heating. (**A**) Examples of raw data corresponding to real-time *ex vivo* metabolism of EA (green line) and ethanol (orange line) production by PTR-MS measurements in presence of OM and heated OM. (**B,C**) For each experiment, the dark grey bar corresponds to the signal monitored by PTR-MS in the control circuit not containing OM. In the experimental circuit, the EA signal (30 µg/L in the gas phase) (**B**) and the ethanol signal (**C**) were recorded in presence of OM and heated OM. Data represent the normalized CPS mean during the last 30 s of the PTR-MS signals measured at the reached plateaux ± SEM. Significant differences are indicated by different letters at level p = 0.05, n ≥ 3 (one-way ANOVA followed by multiple comparison Tukey’s test).

**Figure 5:**
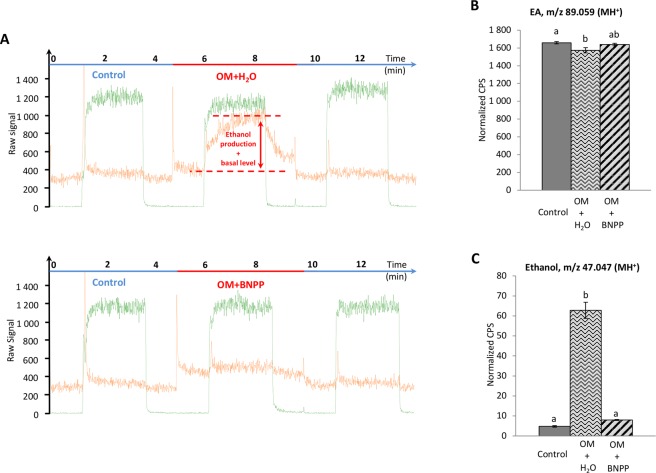
Real-time *ex vivo* OM metabolism of ethyl acetate and ethanol production by PTR-MS measurements: effects of the specific carboxylesterase inhibitor BNPP. (**A**) Examples of raw data corresponding to real-time *ex vivo* metabolism of EA (green line) and ethanol (orange line) production by PTR-MS measurements in presence of OM + H_2_O (100 µL of ultrapure water) and OM + BNPP (100 µL of BNPP at 100 µmol/L in ultrapure water). (**B**,**C**) For each experiment, the dark grey bar corresponds to the signal monitored by PTR-MS in the control circuit not containing OM. In the experimental circuit, the EA signal (30 µg/L in the gas phase) (**B**) and the ethanol signal (**C**) were recorded in presence of OM + H_2_O and OM + BNPP. Data represent the normalized CPS mean during the last 30 s of the PTR-MS signals measured at the reached plateaux ± SEM. Significant differences are indicated by different letters at level p = 0.05, n ≥ 3 (one-way ANOVA followed by multiple comparison Tukey’s test).

In Figs 4 and 5, panel A presents an example of PTR-MS raw data under different experimental conditions to assist with understanding of panels B and C.

Figure 4B shows a significant decrease (−1.17-fold; p < 0.0001) of the EA signal (averaged during the 30 last seconds of the signals measured at the reached plateau) at m/z 89.059 (MH^+^) with continuous exposure to the OM, suggesting EA metabolism. With the heated OM control, the EA signal intensity was slightly lower (−1.04-fold; p = 0.09) than that of the control without the OM, but this difference was not significant (Fig. 4B). This could be due to the partial adsorption of the volatile compound into the heated tissue or incomplete inactivation of the enzymes by the heating procedure. Partial adsorption of EA on the OM cannot be ruled out.

EA metabolism was supported by the appearance of ethanol (m/z 47.049), whose concentration attained a significantly higher intensity (+4.25-fold; p < 0.0001) than the basal level in the control circuit (Fig. 4C). We observed a lower level of ethanol with the heated OM than the basal ethanol level in the control (Fig. 4C), likely due to partial adsorption of ethanol onto the heated OM.

The action of BNPP, a specific inhibitor of carboxylesterase activity^27,28^, on the OM, was assayed (OM + BNPP) and compared with a control consisting of the addition of 100 µL of ultrapure water on the mucosa (OM + H_2_O), where the volume corresponded to the aqueous solution of BNPP used for this enzymatic inhibition experiment. The EA signal for the BNPP assay (OM + BNPP) was not different from the two controls, and no significant effect of BNPP was observed on this odorant under continuous delivery conditions (Fig. 5B). However, BNPP strongly inhibited ethanol production compared with the OM + H_2_O assay (−7.9-fold; p < 0.0001; Fig. 5C). The level of ethanol in the BNPP assay was not significantly different from the basal ethanol level (1.67-fold; p = 0.210). Although *in situ* spontaneous non-enzymatic hydrolysis of EA leading to ethanol production cannot be totally ruled out, previous data^21^ and the different controls used, together with the specific inhibitor effect, support the involvement of carboxylesterases in the olfactory metabolism of EA.

For method validation purposes and to choose the best conditions for EA analysis, additional experiments were performed (see Supplementary Figs S1 and S2). As expected, we found that the synthesis of ethanol was increased when more OM was in the system, corresponding to a higher enzymatic capacity (see Supplementary Fig. S1). Whole OM was chosen for analysis so that a significant level of metabolite (+17-fold; p < 0.0001) could be measured. Additionally, while a concentration of 240 µg/L of EA led to the production of a highly significant level of ethanol (21.04-fold; p < 0.0001; see Supplementary Fig. S2B), we observed no significant decrease in EA in the presence of OM at this concentration (−1.13-fold; p = 0.345; see Supplementary Fig. S2A), likely due to the continuous delivery of EA onto the OM. However, with an EA concentration of 30 µg/L, both the EA decrease (−1.17-fold; p < 0.0001) and ethanol synthesis (+4-fold; p < 0.0001) were significantly different from controls (see Supplementary Fig. S2C,D), explaining why this concentration was chosen.

**Figure 6:**
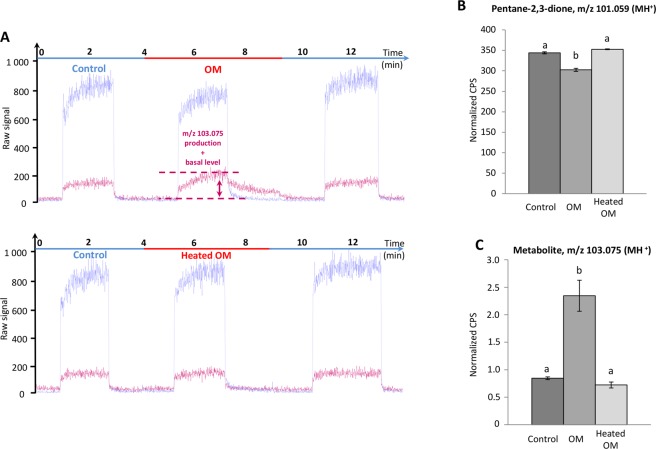
Real-time *ex vivo* OM metabolism of pentane-2,3-dione and production of a metabolite at m/z 103.075 by PTR-MS measurements. (**A**) Examples of raw data corresponding to real-time *ex-vivo* metabolism of pentane-2,3-dione (purple line) and a metabolite at m/z 103.075 (pink line) production by PTR-MS measurements in presence of OM and heated OM. (**B,C**) For each experiment, the dark grey bar corresponds to the signal monitored by PTR-MS in the control circuit not containing OM. In the experimental circuit, the pentane-2,3-dione signal (5 µg/L in the gas phase) (**B**) and the production of a metabolite at m/z 103.075 signal (**C**) were recorded in presence of OM and heated OM. Data represent the normalized CPS mean during the last 30 s of the PTR-MS signals measured at the reached plateaux ± SEM. Significant differences are indicated by different letters at level p = 0.05, n ≥ 3 (one-way ANOVA followed by multiple comparison Tukey’s test).

Finally, considering all the real-time metabolite synthesis measured by the method in this work, we observed that the corresponding signal showed an increasing kinetic profile following OM exposure with the odorant (in addition to the PTR-MS raw data for ethanol, see the data for pentane-2,3-dione; Fig. 6), finally reaching a plateau. This may suggest that the *in situ* synthesis is rapid. Because the first moment of ethanol recording was affected by the presence of a burst of ethanol under the experimental conditions, we proceeded to mathematical extrapolation of the ethanol signal from the slope of the curve (see Supplementary Fig. S3) compared with the same extrapolation for the signal in the presence of the heated OM. The extrapolation suggested that, under the experimental conditions, the appearance of the ethanol metabolite may become significantly different from the enzymatic control (heated OM, p < 0.01) 2.5 seconds after starting continuous delivery of the odorant above the OM.

### Real-time olfactory metabolism of diketones or keto-esters: pentane-2,3-dione, hexane-2,3-dione, hexane-3,4-dione and 2-acetoxybutan-3-one

Following the feasibility assay using EA, subsequent assays were conducted on another family of odorants that is structurally different and *a priori* does not involve the same metabolizing enzymes. Therefore, the enzymatic biotransformations of pentane-2,3-dione, hexane-2,3-dione, hexane-3,4-dione and 2-acetoxybutan-3-one, all of which pertain to the “fatty” sensory perception for humans, were investigated using the developed method.

The pentane-2,3-dione PTR-MS signal was recorded and averaged during the 30 last seconds of signal measurement at the plateau of m/z 101.059 (MH^+^) on continuous delivery (5 µg/L in the gas phase of gas bag B) of the gaseous odorant.

In Fig. 6, panel A presents an example of PTR-MS raw data under different experimental conditions to assist with the understanding of panels B and C.

Figure 6B shows a significant decrease (−1.14-fold; p < 0.0001) in the pentane-2,3-dione signal in the presence of OM compared with the control, suggesting metabolic activity toward the pentane-2,3-dione. With the heated OM, the pentane-2,3-dione signal was not significantly different from the control, suggesting that an enzymatic process was involved in the decrease of the pentane-2,3-dione signal in the presence of intact OM.

Concomitant with the depletion of the MH^+^ signal of pentane-2,3-dione, a signal centered at m/z 103.075 appeared as the only observable metabolite on the PTR-MS spectrum with an intensity significantly higher than that of the basal level control (+2.78-fold; p < 0.0001; Fig. 6C) and in the heated OM assay (+3.25-fold; p < 0.0001) and was also not significantly different from the basal level (Fig. 6C).

**Figure 7:**
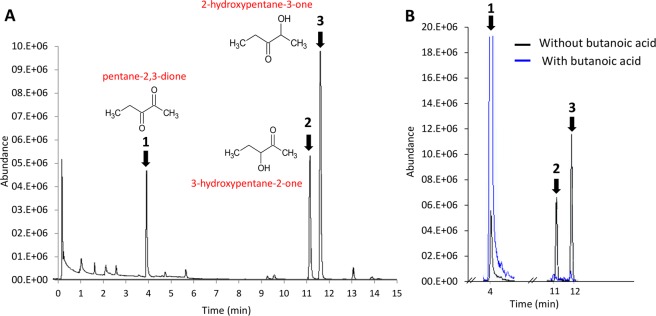
Identification of the metabolites of pentane-2,3-dione produced by OM by headspace gas chromatography/mass spectrometry analysis using SPME fibers and enzymatic inhibitory effect of butanoic acid. The peaks 1, 2 and 3 correspond to pentane-2,3-dione, 3-hydroxypentane-2-one and 2-hydroxypentane-3-one, respectively. GC-MS identification of the metabolites of pentane-2,3-dione (**A**) was realized after SPME extraction of the headspace of a vial containing OM incubated (30 min) with pentane-2,3-dione (1 mg/L in the gas phase). To inhibit the olfactory metabolism of the pentane-2,3-dione, 100 µL of butanoic acid (DCXR enzyme inhibitor) at 100 mM was added on the OM (**B**).

The ion at m/z 103.075 could be the protonated molecular ion MH^+^ of a molecule with a formula C_5_H_10_O_2_ corresponding to a partially reduced form of pentane-2,3-dione C_5_H_8_O_2_. To definitively identify this metabolite, GC-MS analysis was performed after extraction by SPME from the headspace of a vial containing a fresh explant of OM that was in contact with the odorant pentane-2,3-dione (1 mg/L in the gas phase) for 30 minutes. The resulting chromatogram (Fig. 7A) showed, together with some remaining diketone odorant, the production of two volatile metabolites whose specificity was demonstrated by not being present in the control vials containing only the OM or only the odorant, indicating that both the OM and odorant were needed to produce them. Based on their mass spectra and GC linear retention indices (LRIs) and compared with the available data in various databases (Volatile compounds in Food, Nist, Wiley through ACS SciFinder) and the LRI and MS databases of volatile compounds in our laboratory, INRAMass, these two metabolites were unambiguously identified as 3-hydroxypentan-2-one (Fig. 7A, peak 2) and 2-hydroxypentan-3-one (Fig. 7A, peak 3), respectively, in the order of elution. These two positional isomers correspond to the metabolite signal measured at m/z 103.075 by PTR-MS for their MH^+^ ion but could not be distinguished by this technique because of their identical mass. They are both partially reduced forms of the initial diketone.

Pentane-2,3-dione is metabolized into 2-hydroxypentan-3-one by the human airway epithelium through the action of the enzyme dicarbonyl/L-xylulose reductase (DCXR)^30^, and short-chain fatty acids, especially butanoic acid, have a high inhibitory effect on the reductase activity of mammalian DCXR^31^. Additionally, DCXR is expressed in rat olfactory epithelium at higher levels than that in the respiratory epithelium, and these enzymes are involved in pentane-2,3-dione metabolization^32^. In this study, we observed that, in the presence of butanoic acid (Fig. 7B, blue line), the level of pentane-2,3-dione was restored (Fig. 7B, peak 1) compared with the control, and its metabolites, hydroxypentanones, were not produced much (Fig. 7B, peaks 2 and 3), confirming the results obtained by heating the tissue and demonstrating that DXCR is involved in pentane-2,3-dione olfactory metabolism.

Vicinal ketols can undergo isomerization^33^, and, therefore, racemization at their asymmetric center can occur^34^. To address this question for the present pentane-2,3-dione α-hydroxyketone metabolites, a cold on-column injection on a chiral GC column was compared with the heat desorption of an SPME fiber on the same chiral column (see Supplementary Fig. S4). The headspace of a vial containing a fresh OM explant that was in contact for 30 minutes with pentane-2,3-dione was trapped on a Tenax tube that was subsequently desorbed by a solvent for cold on-column injection and on a SPME fiber as previously described. Cold on-column injection on the chiral Lipodex E column showed a 2-hydroxypentan-3-one/3-hydroxypentan-2-one ratio of 4:1 (see Supplementary Fig. S4B), whereas desorption of the SPME fiber in a hot injector showed a ratio of 1.6:1 (see Supplementary Fig. S4A), confirming thermal isomerization had occurred and revealing regioselectivity of the enzymatic reaction in favor of the 2-hydroxy isomer. Moreover, separation on the chiral column revealed a large enantiomeric excess (96:4 for the major 2-hydroxypentan-3-one) and demonstrated that the enzymatic reaction is stereoselective. The exact stereochemistry of the hydroxypentanones was not determined.

Real-time metabolism assays were conducted using hexane-2,3-dione and hexane-3,4-dione and produced similar results (see Supplementary Figs S5 and S6), yielding, respectively, the two metabolites 2-hydroxyhexan-3-one/3-hydroxyhexan-2-one at m/z 117.091 (MH^+^) and 4-hydroxyhexan-3-one at m/z 117.091 (MH^+^). Real-time metabolism assays conducted on 2-acetoxybutan-3-one produced the metabolite diacetyl at m/z 87.0441 (MH^+^; see Supplementary Fig. S7A,B), while GC-MS analysis, measuring the metabolism over 30 minutes, revealed the presence of acetoin, thus suggesting sequential metabolism involving different enzymes (see Supplementary Fig. S7C,D).

## Discussion

It is now clear that olfactory metabolism is involved in the clearance of odorant molecules in the peripheral olfactory system. It is necessary to eliminate the odorant background to maintain the detection sensitivity. However, the ability of this enzymatic mechanism to generate odorant metabolites *in situ* remains largely unknown. To increase the knowledge in this field, we developed a method that continuously delivers an odorant to a fresh explant of rat OM. Thus, the OM was exposed to a slow continuous airflow containing a diluted odorant that mimicked the flow rate of normal rat sniffing^35^. The dispersion of the odorant flow was ensured using a strainer, which allowed better exposure to the whole olfactory mucosa. Connected to a PTR-MS, the system allowed real-time continuous monitoring from the headspace above the explant of both the depletion of the studied odorant and synthesis of its metabolites. To confirm that these records resulted from enzymatic metabolic activity, control experiments were necessary. Therefore, we showed that *ex vivo* odorant uptake by the OM and release of the corresponding volatile metabolites were efficiently inhibited when the tissue had been heated or when an inhibitor was directly applied on the tissue explant a few minutes before signal acquisition.

Regarding odorant metabolism in the OM, *in vitro* studies have been performed on olfactory tissue homogenates and subcellular fractions (microsomes and cytosol) to characterize the activity and kinetic parameters of OMEs toward odorants or other compounds^10,15,18,36–38^. To closer approximate olfactory physiological conditions, *ex vivo* headspace methods have been established to measure odorant metabolism in the vapor phase over OM explants^20,21,39,40^. The original method presented in this work combines the benefits of the *ex vivo* headspace method with real-time monitoring using PTR-MS. Through continuous odorant delivery to the OM, we demonstrated, in real-time, the uptake and conversion of odorants by the OM that involves hydrolytic or reductase enzymatic activity. Moreover, the continuous gaseous delivery of odorant demonstrated the capacity of the OM to efficiently take odorants in charge, even under these *ex vivo* conditions. Interestingly, we recorded a decrease in the odorant simultaneously with the synthesis of its metabolites detectable in the headspace. This last point is particularly interesting regarding the physiologically based pharmacokinetic (PBPK) compartment model of the nasal cavity that was previously proposed^23,41^. Our update relies on evidence that volatile metabolites are released in the OM headspace through the mucus layer proportionally to their air/mucus partition coefficient. This new result highlights the role of air flow and mucus flow in the elimination of volatile metabolites, in addition to blood flow. Desorption of volatile metabolites from the mucus to the air was not considered in the original nasal PBPK models^23,41^.

Our real-time analysis of the release of the odorant metabolites in the headspace showed an increasing synthesis kinetic profile, suggesting rapid metabolism of odorants by the OM. Such a possibility would partially change the current approach of the peripheral olfactory process because metabolites may be considered potential activators of the olfactory receptors. This is supported by recent work in mice, which showed, for the first time, that odorant metabolites can activate odorant receptors^42^. In this work, the authors showed *in vitro* that Cyp1a2 biotransforms acetophenone into the odorant metabolite methyl salicylate, which activates the mouse olfactory receptor 161-2 (MOR161-2). Moreover, after *in vivo* exposure to the metabolite methyl salicylate, *in situ* imaging of the OM showed that MOR161-2 and ribosomal S6 protein, which is phosphorylated in response to neuronal activation, indeed colocalized, attesting to the *in vivo* activation of the olfactory receptor by the metabolite.

Regardless of whether the metabolism occurs in the mucus or OM cells, our results, showing that volatile metabolites can be released into the headspace, support the idea that metabolites can physically contact and thus potentially activate or interact with olfactory receptors located in the membrane of the sensory neuron cilia bathing in the mucus.

EA is a small carboxylic acid ester with a sweet fruity smell that has been widely studied as a solvent or wine component. Our method revealed that ethanol is released by the OM in the receptor vicinity after exposure to EA. As confirmed using the carboxylesterase inhibitor BNPP^28^, the ethanol metabolite resulted from olfactory carboxylesterase activity. These enzymes and their corresponding activities have been previously evidenced in the OM^18,43,44^.

Olfactory metabolism of pentane-2,3-dione, a food-grade diketone aroma presenting fatty sensory characteristics in humans, produced two metabolites: 3-hydroxypentan-2-one and 2-hydroxypentan-3-one. These molecules have been previously identified as aroma compounds in various foodstuffs and beverages where pentane-2,3-dione is ubiquitous, such as dairy products^45–48^, red wine vinegar^49^ and wine^50,51^; they are thought to be produced through the reductive action of *Saccharomyces cerevisiae* on the corresponding 2,3-diketone^52^.

Interestingly, 2-hydroxypentan-3-one, identified in wine, presents a different sensory descriptor in GC-olfactometry (GC-O) than pentane-2,3-dione (burning and alcohol compared with caramel and yogurt, respectively)^51^, whereas 3-hydroxypentan-2-one is described as roasty or toasty in the GC-O of an extract of wine vinegar^49^. Additionally, the metabolites of the other odorants studied in this work (see Supplementary Materials) also showed distinct descriptors from the parent molecule (Table 1).

**Table 1:** Sensory descriptors of the odorants tested and their corresponding metabolites.

Odorant	Metabolites
**Pentane-2**,**3-dione** CAS 600-14-6 **[odor type** = **buttery]** *Sweet, buttery, caramellic, toasted, nutty, marshmallow, molasses nuances – *The Good Scents Company* * Butter, caramel, fruit, sweet – *Volatile Compounds in Food ver 16*.*4*	**2-Hydroxypentan-3-one** CAS 5704-20-1 **[odor type** = **truffle]** *Truffle, earthy, nutty *– The Good Scents Company* *Earth, nut, truffle *– Volatile Compounds in Food ver 16*.*4* *Hay-like, buttery *– Flavor base 10*^*th*^ *Edition*^3^
	**3-Hydroxypentan-2-one** CAS 3142-66-3 **[odor type** = **herbal]** *Herbal, truffle (odor), buttery, creamy, earthy (flavor) – *The Good Scents Company* *Herb, truffle – Volatile Compounds in Food ver 16.4 * Buttery-creamy, caramel, slightly fruity – Flavor base 10^th^ Edition
**Hexane-2**,**3-dione** CAS 3848-24-6 **[odor type** = **buttery]** * Creamy, fruity, toasted, brown caramellic notes *– The Good Scents Company*	**2-Hydroxyhexan-3-one** CAS 54073-43-7 *Fruity, berry *– (Fan et al*., *2012)* *Green, hay-like, sour milk – *(Neuser et al*., *2000)*
	**3-Hydroxyhexan-2-one** CAS 54123-75-0 * Mushroom, earthy – (Neuser *et al*., 2000)
**Hexane-3**,**4-dione** CAS 4437-51-8 **[odor type** = **buttery]** *Buttery, toasted almond, nutty, caramellic (odor) *Caramel, nutty, buttery (flavor) *– The Good Scents Company* *Fat – Volatile Compounds in Food ver 16.4	**4-Hydroxyhexan-3-one** CAS 4984-85-4 *Fruity, somewhat burnt and caramellic, somewhat buttery, caramellic, dairy notes *– Flavor base*
**2-Acetoxybutan-3-one** CAS 4906-24-5 **[odor type** = **fruity]** *Odor, 10%: sweet, creamy, buttery *Odor, 5%: sweet, fruity, estery, chemical, pineapple, apple, banana *Flavor, 100 mg/L: fruity, fleshy, rummy, grape, winey *– The Good Scents Company*	**Diacetyl (butane-2**,**3-dione)** CAS 431-03-8 **[odor type** = **buttery]** *Buttery, sweet, creamy, caramellic, pungent *Odor, 1%: sweet, creamy, buttery, pungent, caramellic * Flavor, 50 100 mg/L: sweet, buttery, creamy, milky *– The Good Scents Company*
	**Acetoin (3-hydroxybutan-2-one)** CAS 513-86-0 **[odor type** = **buttery]** *Odor, 1%: sweet, buttery, creamy, dairy, milky, fatty *Flavor: creamy, dairy, sweet, oily, milky, buttery *– The Good Scents Company*

The use of inhibitors determined the role of the enzyme DCXR in the synthesis of both metabolites. The metabolite 2-hydroxypentan-3-one (but not 3-hydroxypentan-2-one) was recently identified *in vitro* in human cultured normal bronchial/tracheal epithelial cells following pentane-2,3-dione exposure^30^, confirming the role of DCXR in this metabolism. Our results are supported both by the high activity of DCXR in the OM (10 times higher than in the respiratory or the tracheal mucosa) and its localization in the rat OM^32^.

Although some differences exist between rats and humans regarding olfactory metabolism^23^, their OM enzymatic equipment is quite similar^53,54^. Therefore, as in drug metabolism, the rat is a validated model that can be used with our method to screen and characterize odorant volatile metabolites. In this work, we focused on the first enzymatic step of the biotransformation of odorants through catalytic hydrolysis, oxidation or reduction, leading to volatile metabolites. However, many other OMEs catalyze conjugation reactions (with glutathione, sulfate or glucuronic acid) to the odorant itself, or, more often, to the first-step metabolites^9,14,55^. Because such reactions synthesize highly hydrophilic metabolites (undetectable by PTR-MS), they may not have a signaling function, but this cannot be ruled out according to the versatility of olfactory reception.

In this study, we made steps toward a spatially refined picture of real-time *in situ* odorant metabolism. The abundance and diversity of OMEs in the OM control odorant bioavailability and allow the potential conversion of nearly infinite odorants. Our method allows the systematic screening of volatile metabolites resulting from odorant metabolism in a validated animal model. To directly address whether metabolites contribute to the olfactory code, it is first necessary to extend the method to shorter timescales. Minimizing the dead volumes in the circuits, particularly using a low-dead volume 6-way valve, could improve the experimental timescale. Further investigation is needed to characterize the sensory characteristics of odorant metabolites in humans. It is important to access the detection thresholds compared with native odorant thresholds measured with the same panel and to learn about their involvement and behavior in combinatorial coding and competitive interactions with EMO. This will certainly improve our approach to the peripheral olfactory system in the context of physiological or pathophysiological conditions. Additionally, the characterization of odorant metabolites with new or enhanced sensory properties may have applications in the aroma industry.

## Methods

### Chemicals

All the chemicals ethyl acetate (EA; CAS#141-78-6), pentane-2,3-dione (CAS#600-14-6), hexane-2,3-dione (CAS#3848-24-6), hexane-3,4-dione (CAS#4437-51-8), 3-oxobutan-2-yl acetate (2-acetoxybutan-3-one, CAS# 4906-24-5), butanoic acid (CAS#107-92-6), bis (4-nitrophenyl) phosphoric acid (BNPP; CAS#645-15-8) and diethyl ether (CAS#60-29-7) were purchased from Sigma Aldrich (Sigma Aldrich, St Quentin Fallavier, France).

### Animals

Male Wistar rats were bred and hosted in an exempt organisms pathogen -specific (E.O.P.S.) animal area of the animal facility of the plateforme de zootechnie (University of Dijon) and kept Under a constant 12 h light - 12 h dark cycles, 50% of humidity, temperature 22 °C. They were raised in ventilated rack cages, with enrichment cell sizzle (SAFE, Augy 89) fed ad libitum with standard food from SAFE (A03-10 pour breeding, next A04-10). After decapitation of the animals, careful OM dissection was performed to avoid contamination with respiratory epithelium. The freshly removed OMs were immediately placed convolution upwards in the suitable vessel for on-line measurements (*vide infra*) or in 20 mL vials sealed with a Teflon-lined stopcock for adsorptive headspace analyses (SPME and Tenax trapping). Time between dissection and analysis was kept as short as possible (ca. 10 min.) to reduce potential loss of metabolic activity. We used a total of 60 rats for *ex vivo* measurement. The local, institutional and national guidelines and regulation regarding the applied methods, the care and experimental uses of the animals were followed. Thus, all experimental protocols were conducted in accordance with ethical rules enforced by French law, and were approved by the local Ethical Committee of the University of Burgundy (Comité d’Ethique de l′Expérimentation Animale Grand Campus Dijon; C2EA grand campus Dijon N°105), and by the French Ministère de l′Education Nationale, de l′Enseignement Supérieur et de la Recherche under the no. 3504.

### PTR-ToF-MS

All the experiments were conducted with a proton transfer reaction-mass spectrometer (PTR-MS) instrument equipped with a Time-of-flight (Tof) analyzer (PTR-ToF 8000, Ionicon Analytik, Innsbruck, Austria). Data were recorded with the TOF-DAQ software in the range m/z 0 to 250 at one spectrum per 0.499 second. The mass resolution varied from 3500 to 4500 along the scanned mass range. Mass axis calibration and calculation of peak areas were done with the software PTR-MS Viewer ver. 3.1.0.28. The primary ions m/z = 19.018 [H_3_O^+^] and m/z = 37.028 [(H_2_O)_2_H^+^] were systematically monitored following their respective ^18^O isotopic contributions at 21.022 [H_3_^18^O^+^] and m/z 39.032 [H_2_O^18^OH_3_^+^] to check instrument performances and to detect cluster formation. The mass calibration was realized following the peaks of known ions (H_3_^18^O^+^, m/z = 21.022086; NO^+^, m/z = 29.997440; protonated acetone, m/z = 59.049141) present at any time in spectra. The intra-measurement variations of the H_3_O^+^ primary ion intensity (monitored on H_3_^18^O^+^) did not exceed 5–6%, and no depletion of the main reactant ion was noticeable. During the acquisition we focused on specific masses: m/z = 47.047 (protonated ethanol) and m/z = 89.059 (protonated EA) for experiments using EA, m/z = 101.059 (protonated pentane-2,3-dione) for the investigation of pentane-2,3-dione, m/z = 115.075 (protonated hexane-2,3-dione) for the investigation of hexane-2,3-dione, m/z = 115.075 (protonated hexane-3,4-dione) for the investigation of hexane-3,4-dione and m/z = 131.070 (protonated 2-acetoxybutan-3-one) for the investigation of 2-acetoxybutan-3-one. All the analyses were carried out at a drift-tube pressure of 2.3 mbar, a drift-tube temperature of 80 °C and a drift-tube voltage of 486 V giving an electric field strength to number density ratio E/N of 110 Td (1 Td = 10^−17^ cm^2^.V) and H_3_O^+^ as reagent ion. All the spectra were background subtracted using the background signal (see Fig. 3) before the odorant delivery. The data were corrected for transmission and expressed in normalized CPS using primary ions H_3_O^+^ and (H_2_O)_2_H^+^ to account for primary ions fluctuations.

### On-line measurements

The developed instrumentation consisted in an oven-thermostated (30 °C) two-independent circuits arrangement implemented from a 6-way valve (Rheodyne, Model 7060 selection valve) whose end was connected to the PTR-MS instrument, allowing odorant delivery alternatively in both circuits (Figs 1 and 2). Each identical circuit consisted in PEEK capillary tubing of suitable length (1 mm internal diameter, 255 cm length, 1/16 in. external diameter) and included a glassware trap (13.2 cm length, 4.6 mL) hermetically closed with a Rotulex® spherical joint and clip. The trap in the experimental circuit received the freshly dissected rat OMs, the other one serving entirely as a control circuit. A stainless-still strainer was installed at the entrance of each trap to spread out the air flow. The complete assembly was connected to the PTR-MS instrument via a PEEK capillary (1 mm internal diameter, 1 m length, 1/16 in external diameter) inserted in a heated transfer line maintained at 110 °C. The flow rate entering the PTR-MS instrument was fixed at 160 mL/min. The gas flows were delivered to the system from Tedlar® gas bags (#GSTP016-1818S with screw cap, Jensen Inert Products) situated outside the oven, at the temperature of the air-conditioned room (22 ± 1 °C) where the PTR-MS instrument was operated. The first one (gas bag A in Fig. 1) contained 250 mL of ultrapure water (milli-Q® system, Millipore, Molsheim France) and was inflated to 15 L with zero-air. This allowed delivery of humidified air through the system to the PTR-MS instrument with constant moisture content. A similar bag was connected to the entrance of the PTR-MS transfer line via a 3-way Luer valve which allowed isolating the system when needed while humidified zero-air was admitted continuously to the instrument. The second one (gas bag B in Fig. 1) also inflated with zero-air to 15 L contained 250 mL of an aqueous solution of an odorant. The air-water partition coefficients of the odorants were measured using the method developed for EA by Faure *et al*.^20^. Their values (Kaw = 12.5 10^−3^ for EA; Kaw = 1.33 10^−3^ for pentane-2,3-dione; Kaw = 4.06 10^−3^ for hexane-2,3-dione; Kaw = 3.40 10^−3^ for hexan-3,4-dione, and Kaw = 7.12 10^−5^ for 2-acetoxybutan-3-one) allowed to determine their respective concentrations in aqueous and gas phases (30 and 240 µg/L) for EA, 5 µg/L for pentane-2,3-dione, 5 and 50 µg/L for hexane-2,3-dione and hexane-3,4-dione and 1 µg/L for 2-acetoxybutan-3-one). The gas bags were prepared the day before the experimentation to ensure headspace equilibrium. Successive fine and shut-off valves placed in the odorant transfer line allowed to adjust the proportion of odorant in the zero-air flow coming from gas bag A (Fig. 1). In a typical experiment a fresh rat OM explant was placed in the glass trap of the experimental circuit. After starting signal acquisition, the system was connected to the PTR-MS transfer line on rotating the entrance 3-way valve. Humidified zero-air from the gas bag A was first flowed at 160 mL/min in the control circuit to obtain the background signal level (see Fig. 2). After one minute, the shut-off valve was opened during two minutes to introduce gaseous odorant in the control circuit. One minute after closing the shut-off valve to recover the background signal level with humidified zero-air in the control circuit, the 6-way valve was switched from the control circuit to the experimental circuit during one minute. This allowed to obtain the background level of the signal in the experimental circuit (see Fig. 2). Without interrupting the acquisition, the shut-off valve was opened during two minutes to introduce gaseous odorant in the experimental circuit. One minute after closing the shut-off valve, the 6-way valve was switched from the experimental circuit to the control during one minute. To finish, the shut-off valve was opened during two minutes in the control circuit to obtain another control. Between each acquisition, the experimental gas trap was cleaned with ultrapure water and the two circuits were dried and cleaned with zero-air to avoid the persistence of odorant molecules.

The system development and controls were realized with EA. Concentration of EA in gaseous phase of the gas bag was 30 µg/L. Different controls were done. We first controlled that the two circuits were identical by measuring the flow rate and the signal of EA in each circuit. The control circuit without OM flowed with a constant amount of humidified zero-air or odorant allowed measuring the odorant reference signal level and the basal signal at the m/z value of the known metabolite (see Fig. 3). The same procedure was applied for all the other odorants. Secondly, in order to denaturate the enzymes and thus to confirm the enzymatic nature of the reaction, OM was previously heated at 80 °C for 15 min and cooled down at room temperature prior to the analysis. In order to reduce the number of rats sacrificed, heated mucosae were beforehand used in experimental conditions. It was previously checked that the signals obtained with OM heated after being used was not different from the signals obtained with a heated OM from a fresh explant. Thirdly, only a 10% decrease of the signal were recorded over 20 minutes of odorant exposure attesting for the capacity of the OM to continuously metabolized EA. Finally, the absence of any significant intrinsic metabolism was checked in the experimental circuit containing only OM flowed with humidified zero-air. Then, to confirm the nature of the enzyme involved in the metabolization of EA (carboxylesterase), 100 µL of Bis(4-nitrophenyl) phosphate (BNPP), a carboxylesterase inhibitor^28^ at 100 µmol/L in ultrapure water was deposited on the dissected OM during 5 minutes before acquisition. As a control, the same experimentation was done with 100 µL of ultrapure water. The last control was to demonstrate the enzymatic implication by comparing ethanol signals (EA metabolite) in presence of different enzymatic concentrations. By increasing the amount of OM, we increased the enzymatic capacity. Various OM quantities were tested: half an OM (0.5 OM), whole OM (OM) and one OM and a half (1.5 OM).

### PTR-ToF-MS data analysis

Results are expressed as mean of mass spectrum recording during the 30 last seconds at the odorant delivery plateau (before closing the shut-off valve). All the spectra were background subtracted by the mass spectra averaged on 30 seconds before opening the shut-off valve (without odorant) (see Fig. 3). The ^18^O isotopologues of pentane-2,3-dione found at m/z 103.064 and of the hexanediones found at m/z 117.080, not being separated from the hydroxy metabolite signals at the operating resolution of the instrument, have been considered and their calculated intensities subtracted from the respective metabolite signals. For each acquisition, results of the two control circuit plateau signals were averaged. For statistical analyses, data were averaged on available replicates and analysed using the one-way ANOVA followed by multiple comparison Tuckey’s test. Data are expressed as means ± SEM (Standard Error of the Mean). As calibration curves were not produced, the data were expressed in normalized CPS using primary ions H_3_O^+^ and (H_2_O)_2_H^+^.

### SPME-GC-MS

To identify the enzymatic metabolites produced in presence of OM, Gas Chromatography/Mass Spectrometry (GC-MS) (Agilent MSD 5973 N GC/MS) analyses of the headspace close to the OM were realized. The use of 3 phases (Divinylbenzene/Carboxen/Polydimethylsiloxane) Solid Phase Micro-Extraction (SPME) fiber allows to capture the maximum amount of metabolites in the headspace of the OM. To analyze the maximum of potential metabolites, a temperature gradient of 5 °C/min from 40 °C to 240 °C was set to the GC-MS with a 1.5 ml/min constant flow. A DB-WAX capillary column (30 m × 0.32 mm, film thickness 0.5 µm, J&W Scientific, Folsom, CA, USA) was generally used except for the thermal isomerization assay where a Lipodex E capillary column (25 m × 0.25 mm, Macherey Nagel, Germany) was used. The mass spectrometer scanned the ion mass fragments (m/z) from 29 to 350. The ion source was set at 230 °C and the transfer line at 250 °C. Helium was used as the carrier gas at a linear velocity of 44 cm/s. Freshly dissected rat olfactory mucosa was placed convolution upwards into a 20 mL vial sealed with a Teflon-lined stopcock. 500 µL of gaseous EA at 40 mg/L was injected with an automatic gaseous syringe in the headspace above the OM to obtain the final concentration of 1 mg/L in the vial. After 30 minutes at 37 °C the SPME fiber was disposed in the vial close to the OM during 10 minutes to adsorb volatile molecules present in the headspace. Then the fiber was recovered and placed into the GC-MS injector set to 240 °C and desorbed for a 5 min sequence in splitless mode. In parallel controls containing only OM without odorant were realized. We similarly tested a shorter exposure time (only 5 minutes) between the OM and the odorant before the introduction of SPME fiber. Similarly, these experimentations were realized with pentane-2,3-dione at 1 mg/L, hexane-2,3-dione at 1 mg/L, hexane-3,4-dione at 1 mg/L and 2-acetoxybutan-3-one at 0.1 mg/L in the vial. We control the Dicarbonyl/L-xylulose reductase (DCXR) activity with injection of 100 µl of the dicarbonyl reductase inhibitor butanoic acid at 100 mM on OM.

### SPME-GC-MS data analysis

The identification of the metabolites was carried out by comparison of their mass spectra with those of NIST08 library, in our INRAmass library, Wiley 138 database and through ACS SciFinder and also by comparing their linear retention indexes (LRI) with those of Flavornet database. LRIs of the compounds were calculated using a series of alkanes (C10 to C30) injected in the same chromatographic conditions. All data were analyzed with the MSD Chemstation software (Agilent).

### Tenax-GC-MS

To evaluate thermal isomerization of pentane-2,3-dione metabolites hydroxyketones, cold on-column injection GC-MS analyses of the headspace close to the OM were realized. After injection of pentane-2,3-dione in the headspace above the OM to obtain the final concentration of 1 mg/L and 30 minutes incubation at 37 °C, metabolites were trapped on a 6.00 mm outside diameter adsorbent tube packed with Tenax® TA (Gerstel) through the pre-drilled Teflon-lined septum of the 20 mL vial containing the OM, and metabolites were trapped by a 10 mL/min airflow intake during 5 minutes. Metabolites desorption from the Tenax trap was realized by using 3 mL of diethyl ether followed by a concentration step using a slight flow of nitrogen. The concentrated metabolites were resolubilized in 5 µL of diethyl ether and 1 µL was cold on-column injected (cold on-column adaptor for the Gerstel CIS injector) on a chiral Lipodex E column (25 m × 0.25 mm, Macherey Nagel, Germany). GC-MS parameters and data analysis were identical to those of SPME-GC-MS previously described.

## Supplementary information

Supplementary dataset
