# Publisher Correction: *Ex vivo* real-time monitoring of volatile metabolites resulting from nasal odorant metabolism

**DOI:** 10.1038/s41598-019-44134-1

**Published:** 2019-05-28

**Authors:** Aline Robert-Hazotte, Rachel Schoumacker, Etienne Semon, Loïc Briand, Elisabeth Guichard, Jean-Luc Le Quéré, Philippe Faure, Jean-Marie Heydel

**Affiliations:** Centre des Sciences du Goût et de l’Alimentation, UMR 6265 CNRS/1324 INRA/Université de Bourgogne Franche-Comté, 9 boulevard Jeanne d’Arc, F-21000 Dijon, France

Correction to: *Scientific Reports* 10.1038/s41598-019-39404-x, published online 21 February 2019

The original version of this Article was published with an incorrect version of the Supplementary Information file due to a technical error. This has now been corrected in the HTML; the PDF version of the paper was correct from the time of publication.

