# Supplementary material for: Ex vivo real-time monitoring of volatile metabolites resulting from nasal odorant metabolism
Source: Sci Rep. 2019 Feb 21;9:2492. doi: 10.1038/s41598-019-39404-x (PMC6385289; doi:10.1038/s41598-019-39404-x)
Supplement: Supplementary file 1 — Supplementary dataset [file 41598_2019_39404_MOESM1_ESM.pdf]

Ex-vivo real-time monitoring of volatile metabolites resulting from nasal odorant metabolism

Aline Robert-Hazotte, Rachel Schoumacker, Etienne Semon, Loïc Briand, Elisabeth Guichard, Jean-Luc Le Quéré, Philippe Faure, Jean-Marie Heydel.

In the following figures, PTR-Tof-MS (see Methods) is the full name of the technique used but has been abbreviated to its common name of PTR-MS.

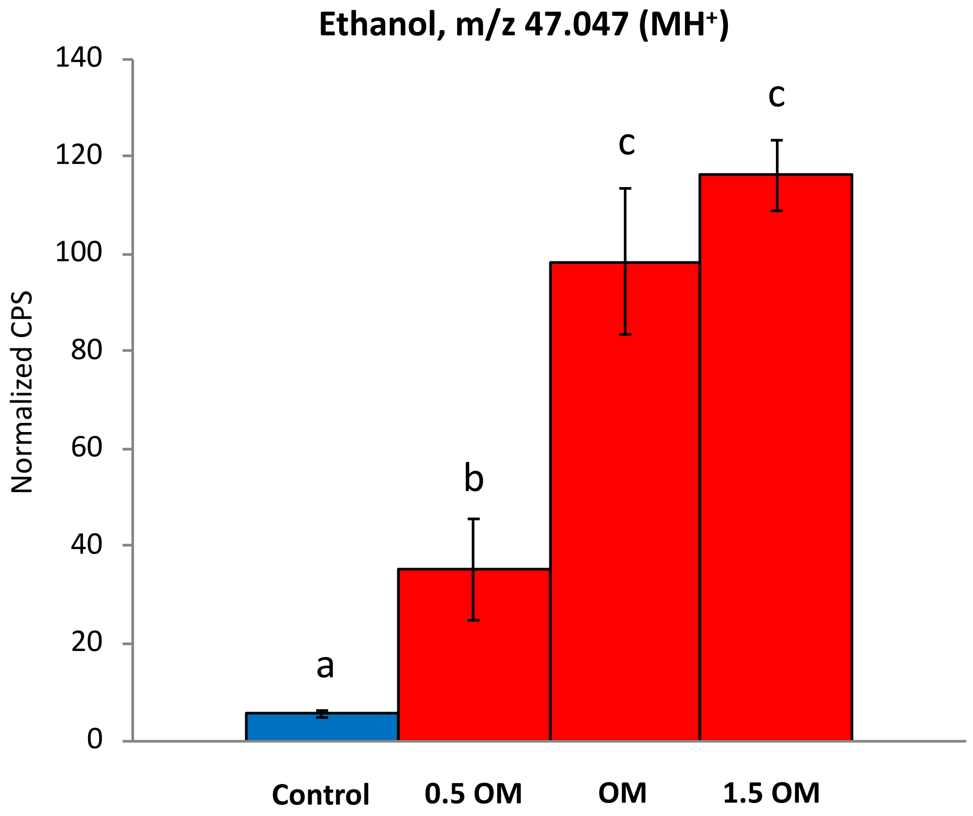

Figure S1– Effect of variations of OM amount on ethyl acetate olfactory metabolism: ethanol measurements by PTR-MS.

The blue bar corresponds to the ethanol signal monitored by PTR-MS in the control circuit not containing OM. In the experimental circuit, the apparition of ethanol was monitored by the real time *ex vivo* PTR-MS method during the continuous delivery of ethyl acetate (30 µg/L in the gas phase). Since the OM is separated into two hemi-mucosa in rat, ethanol signal was measured in presence of one hemi-mucosa (0.5 OM), two hemi-mucosa (OM) and three hemi-mucosa (1.5 OM) in the experimental circuit. Data represent the normalized CPS mean during the last 30 s of the PTR-MS signals measured at the reached plateaux ± SEM. Significant differences are indicated by different letters at level p=0.05, n ≥ 3 (One way ANOVA followed by multiple comparison Dunnett't test).

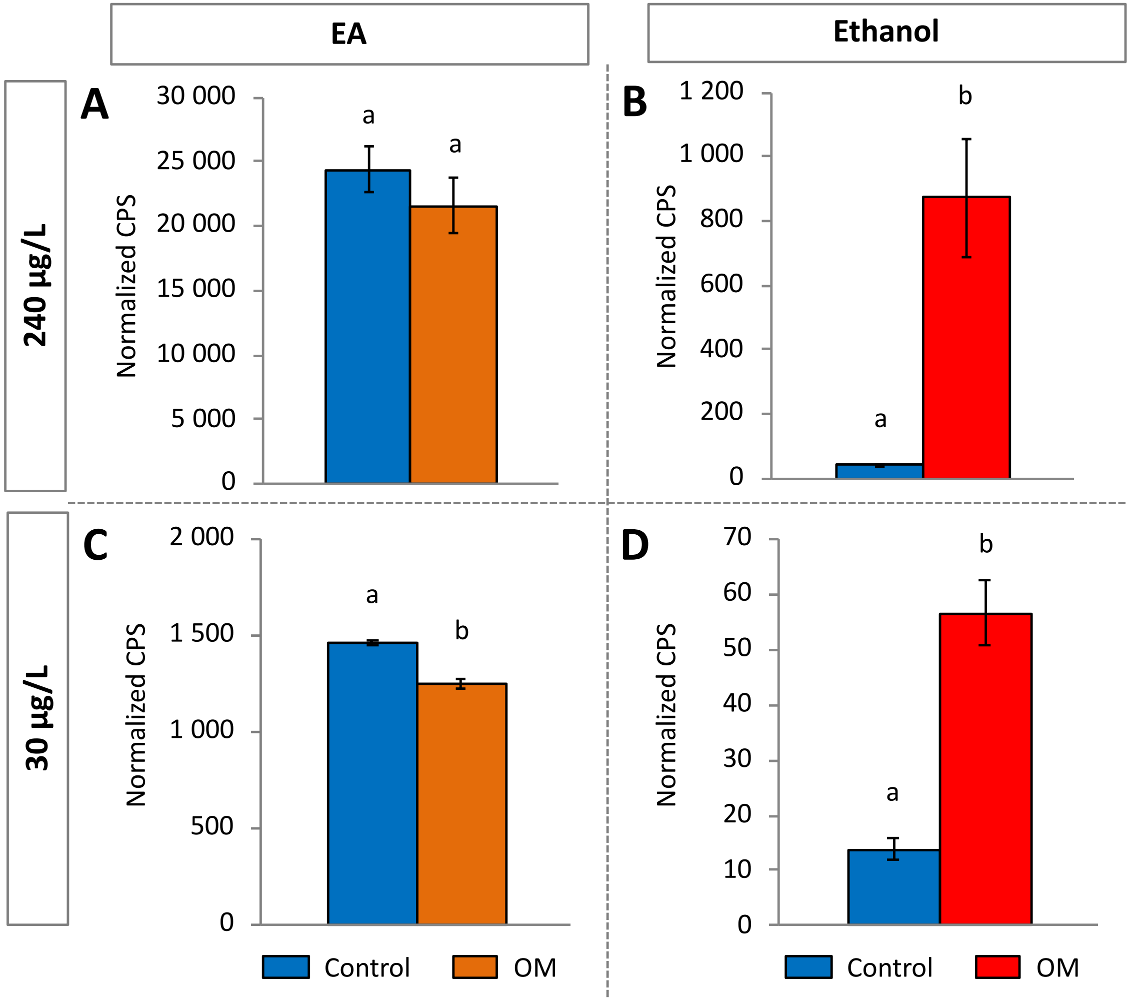

Figure S2 – Effect of variations of ethyl acetate amount on its olfactory metabolism: ethyl acetate and ethanol measurements by PTR-MS.
For each experiment, the blue bar corresponds by PTR-MS in the control circuit not containing OM .The decrease of ethyl acetate signal was measured either in presence of 240 µg/L (**A**) or 30 µg/L (**C**) of ethyl acetate in the gas bag by PTR-MS in the experimental circuit. The increase of ethanol signal was measured either in presence of 240 µg/L (**B**) or 30 µg/L (**D**) of ethyl acetate in the gas bag by PTR-MS in the experimental circuit. Data represent the normalized CPS mean during the last 30 s of the PTR-MS signals measured at the reached plateaux ± SEM. Significant differences are indicated by different letters at level p=0.05, n ≥ 5 (One way ANOVA followed by multiple comparison Dunnett't test).

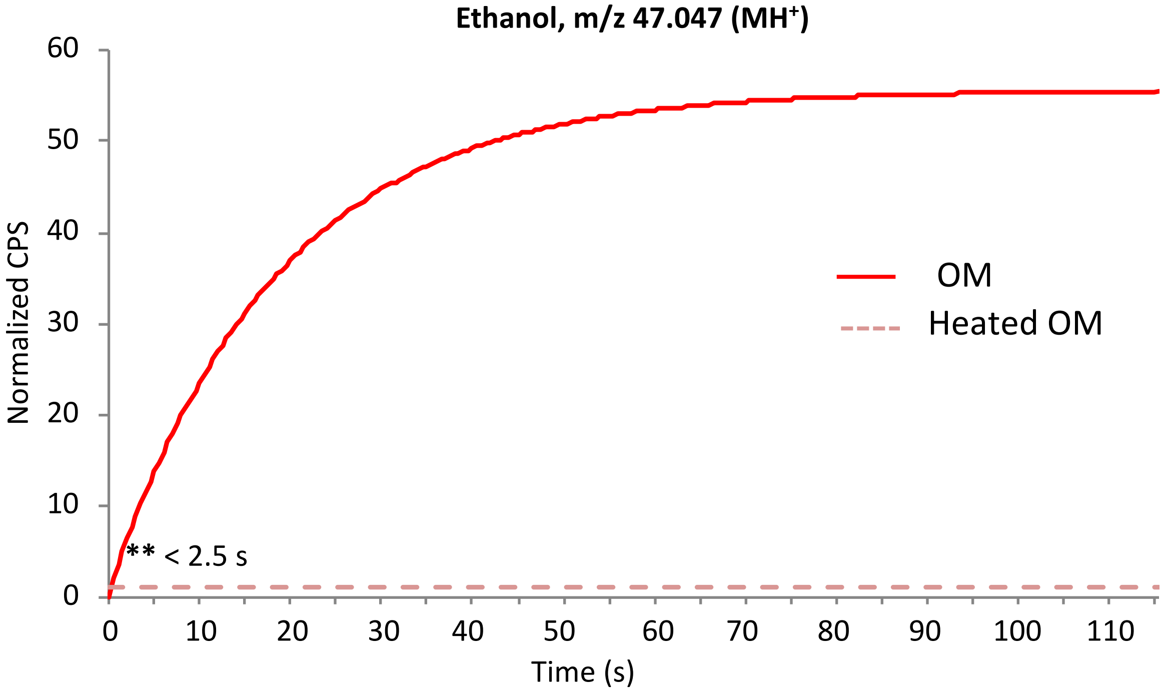

Figure S3 – Smoothed kinetics of the *ex vivo* ethanol production by PTR-MS: comparison between OM and heated OM. The two curves correspond to the smoothed kinetics obtained by mathematical extrapolation of the ethanol production signal from the slope of the curves monitored by PTR-MS with OM or heated OM. Smoothed kinetics of the *ex vivo* ethanol production was measured during 115 s in the experimental circuit containing the OM (solid red line) and was compared with the heated OM (hatched red line). Data represent the mean ± SEM from 3 independent assays. The significant differences are noted **$p<0.01$, (Student's T-test) for a comparison with OM and heated OM.

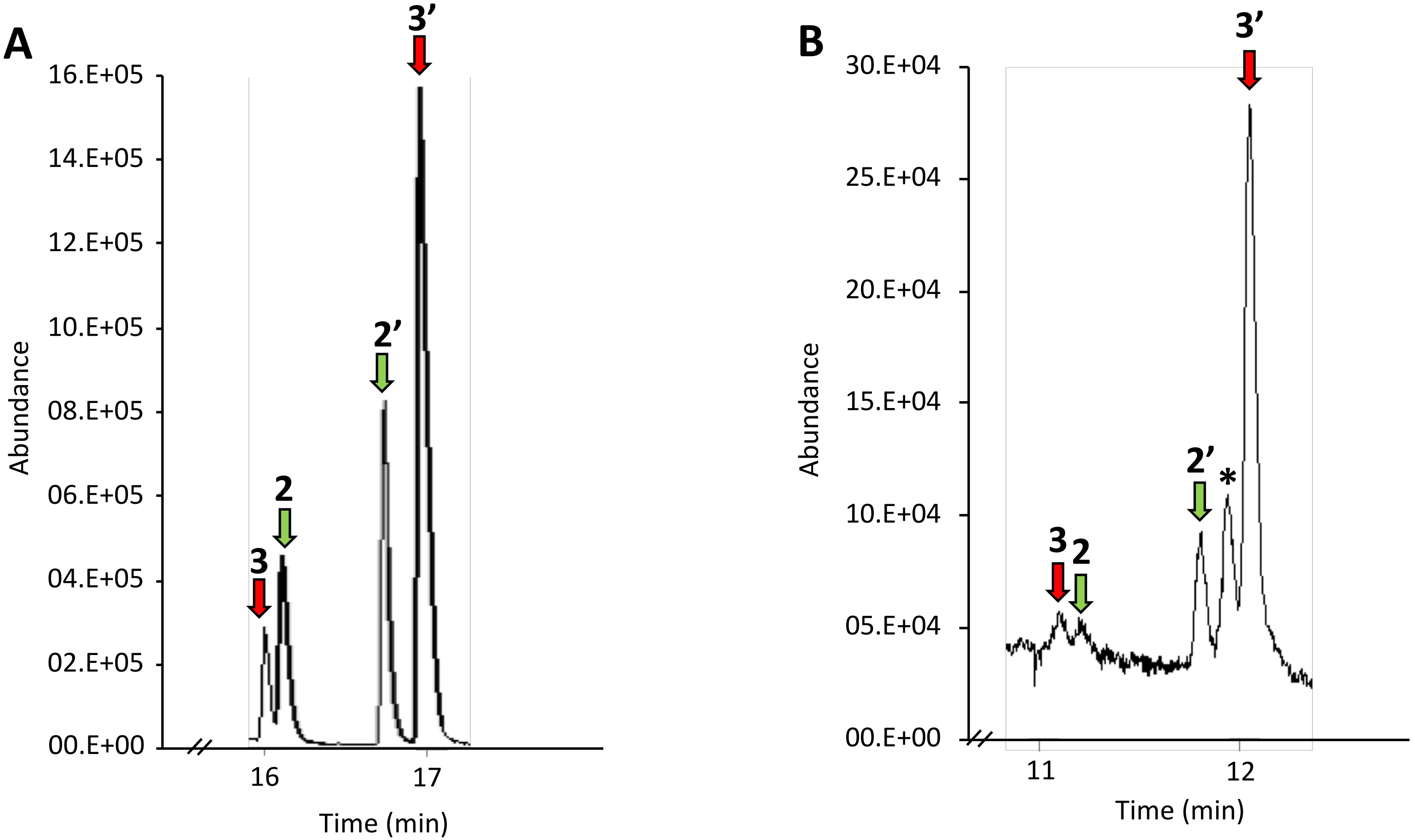

Figure S4 – Evaluation of thermal isomerization of pentane-2,3-dione metabolites hydroxyketones produced by OM by Headspace gas chromatography / mass spectrometry analysis using a chiral column.
The peaks **2** and **2'** correspond to stereoisomers of 3-hydroxypentane-2-one and the peaks **3** and **3'** correspond to stereoisomers of 2-hydroxypentane-3-one, respectively. * is an impurity.
Evaluation of thermal isomerization was realized after extraction of the headspace of a vial containing OM incubated (30 min) with pentane-2,3-dione (1 mg/L in the gas phase) using a SPME fiber desorbed in the heated GC-MS injector at 240°C (**A**) or using a TENAX tube subsequently desorbed by solvent for cold on-column injection (**B**).

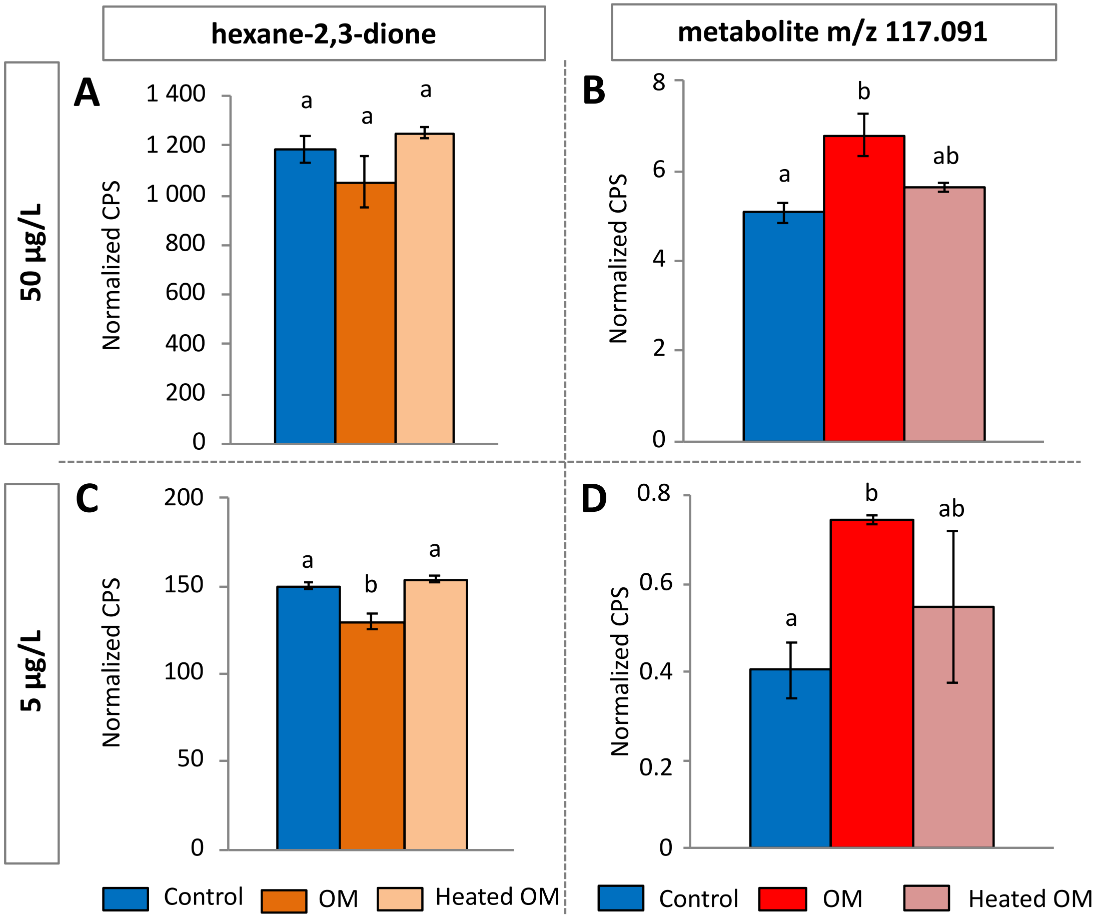

Figure S5 – Effect of variations of hexane-2,3-dione amount on its olfactory metabolism : hexane-2,3-dione and metabolite at m/z 117.091 measurements by PTR-MS.
For each experiment, the blue bar corresponds to the signal monitored by PTR-MS in the control circuit not containing OM. Variations of hexane-2,3-dione signal was measured either in presence of 50 µg/L (**A**) or 5 µg/L (**C**) of hexane-2,3-dione in the gas bag on a fresh explant OM and a heated OM by PTR-MS in the experimental circuit. The increase of metabolite at m/z 117.091 signal was measured either in presence of 50 µg/L (**B**) or 5 µg/L (**D**) of hexane-2,3-dione in the gas bag on a fresh explant OM and a heated OM by PTR-MS in the experimental circuit. Data represent the normalized CPS mean during the last 30 s of the PTR-MS signals measured at the reached plateaux ± SEM. Significant differences are indicated by different letters at level p=0.05, n ≥ 3 (One way ANOVA followed by multiple comparison Dunnett't test).

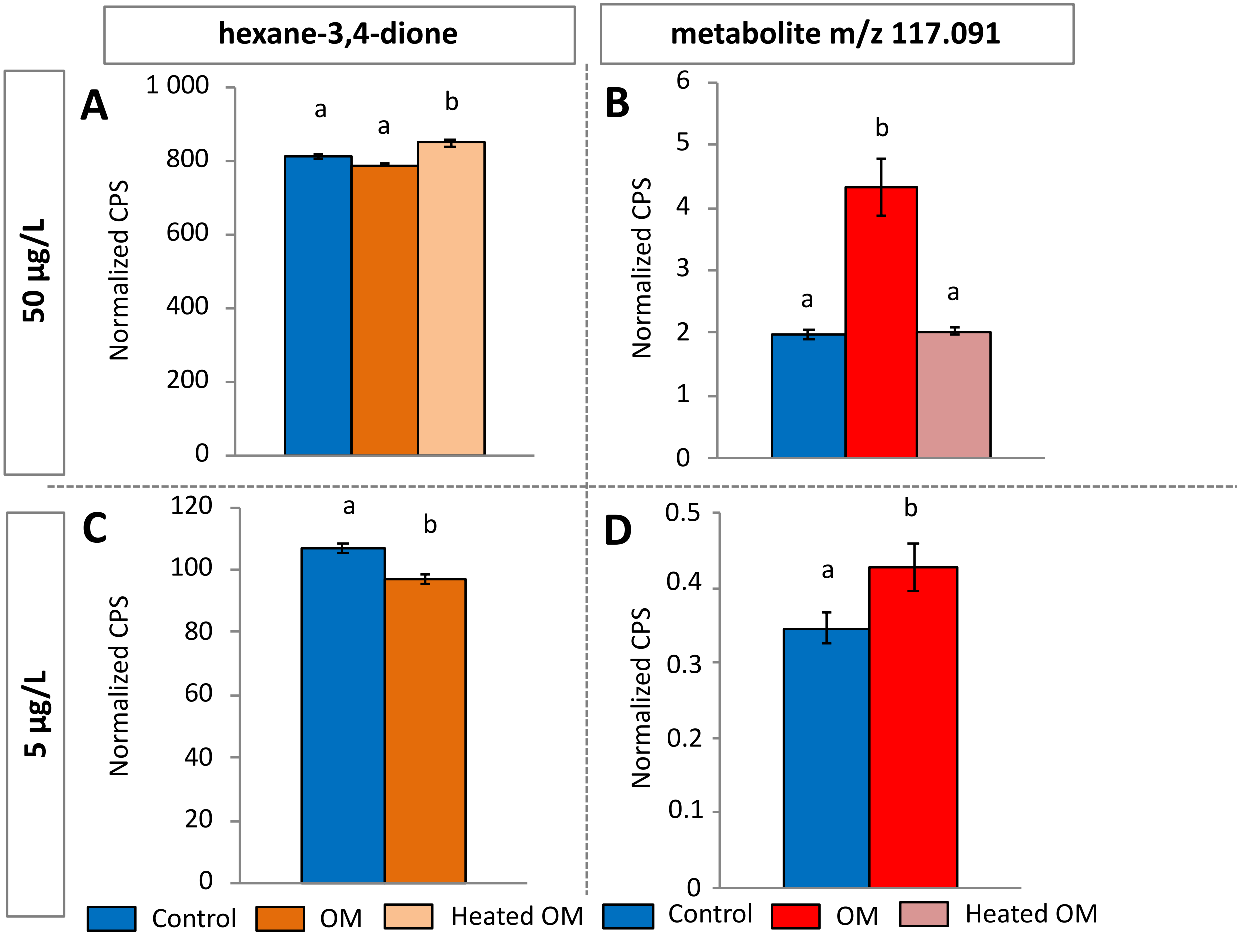

Figure S6 – Effect of variations of hexane-3,4-dione amount on its olfactory metabolism : hexane-3,4-dione and metabolite at m/z 117.091 measurements by PTR-MS.

For each experiment, the blue bar corresponds to the signal monitored by PTR-MS in the control circuit not containing OM. Variations of hexane-3,4-dione signal was measured either in presence of 50 µg/L (**A**) or 5 µg/L (**C**) of hexane-3,4-dione in the gas bag on a fresh explant OM and a heated OM (only for 50µg/L) by PTR-MS in the experimental circuit. The increase of metabolite at m/z 117.091 signal was measured either in presence of 50 µg/L (**B**) or 5 µg/L (**D**) of hexane-3,4-dione in the gas bag on a fresh explant OM and a heated OM (only for 50µg/L) by PTR-MS in the experimental circuit. Data represent the normalized CPS mean during the last 30 s of the PTR-MS signals measured at the reached plateaux ± SEM. Significant differences are indicated by different letters at level p=0.05, n ≥ 3 (One way ANOVA followed by multiple comparison Dunnett't test).

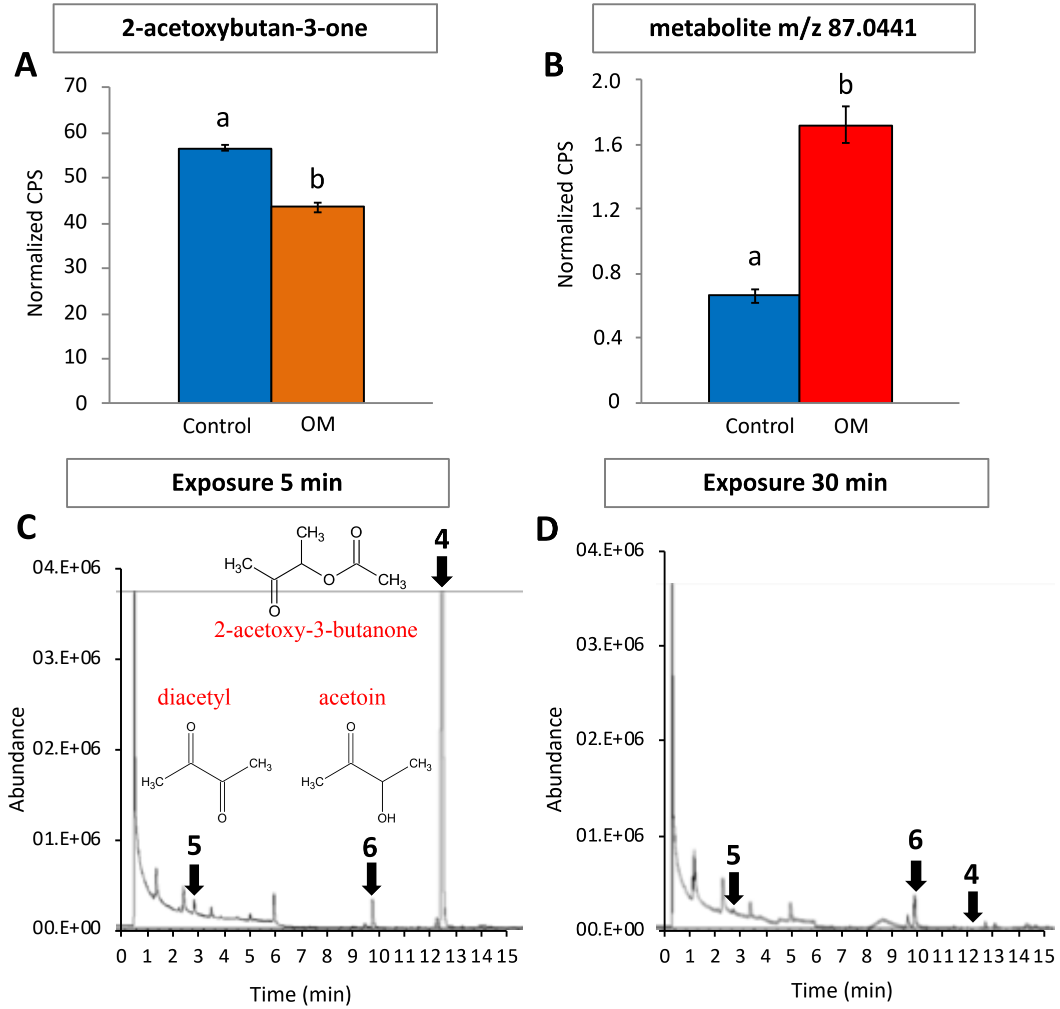

Figure S7– Real time *ex vivo* olfactory metabolism of 2-acetoxybutan-3-one and production of a metabolite at m/z 87.0441 by PTR-MS measurements and identification of the metabolite by headspace gas chromatography / mass spectrometry analysis using SPME fibers.

Blue bar corresponds to the signal monitored by PTR-MS in the control circuit not containing OM. The variations of the signal of 2-acetoxybutan-3-one (**A**) and a metabolite at m/z 87.0441 (**B**) was measured by PTR-MS in the experimental circuit containing OM. Data represent the normalized CPS mean during the last 30 s of the PTR-MS signals measured at the reached plateaux ± SEM. Significant differences are indicated by different letters at level p=0.05, n ≥ 3 (One way ANOVA followed by multiple comparison Dunnett't test). The peaks **4**, **5** and **6** correspond to 2-acetoxy-3-butanone, diacetyl and acetoin, respectively. GC-MS analysis of the metabolites was realized after SPME extraction of the headspace of a vial containing OM incubated 5 min (**C**) or 30 min (**D**) with 2-acetoxybutan-3-one (0.1 µg/L in the gas phase).
